# Novel Pathways for Ameliorating the Fitness Cost of Gentamicin Resistant Small Colony Variants

**DOI:** 10.3389/fmicb.2016.01866

**Published:** 2016-11-22

**Authors:** Martin Vestergaard, Wilhelm Paulander, Bingfeng Leng, Jesper B. Nielsen, Henrik T. Westh, Hanne Ingmer

**Affiliations:** ^1^Department of Veterinary Disease Biology, Faculty of Health and Medical Sciences, University of CopenhagenFrederiksberg, Denmark; ^2^MRSA Knowledge Center, Department of Clinical Microbiology, Hvidovre HospitalHvidovre, Denmark

**Keywords:** *Staphylococcus aureus*, evolution, gentamicin, resistance, small colony variants, electron transport chain

## Abstract

Small colony variants (SCVs) of the human pathogen *Staphylococcus aureus* are associated with persistent infections. Phenotypically, SCVs are characterized by slow growth and they can arise upon interruption of the electron transport chain that consequently reduce membrane potential and thereby limit uptake of aminoglycosides (e.g., gentamicin). In this study, we have examined the pathways by which the fitness cost of SCVs can be ameliorated. Five gentamicin resistant SCVs derived from *S. aureus* JE2 were independently selected on agar plates supplemented with gentamicin. The SCVs carried mutations in the menaquinone and hemin biosynthesis pathways, which caused a significant reduction in exponential growth rates relative to wild type (WT; 0.59–0.72) and reduced membrane potentials. Fifty independent lineages of the low-fitness, resistant mutants were serially passaged for up to 500 generations with or without sub-lethal concentrations of gentamicin. Amelioration of the fitness cost followed three evolutionary trajectories and was dependent on the initial mutation type (point mutation vs. deletion) and the passage condition (absence or presence of gentamicin). For SCVs evolved in the absence of gentamicin, 12 out of 15 lineages derived from SCVs with point mutations acquired intra-codonic suppressor mutations restoring membrane potential, growth rate, gentamicin susceptibility and colony size to WT levels. For the SCVs carrying deletions, all lineages enhanced fitness independent of membrane potential restoration without alterations in gentamicin resistance levels. By whole genome sequencing, we identified compensatory mutations in genes related to the σ^B^ stress response (7 out of 10 lineages). Inactivation of *rpoF* that encode for the alternative sigma factor SigB (σ^B^) partially restored fitness of SCVs. For all lineages passaged in the presence of gentamicin, fitness compensation via membrane potential restoration was suppressed, however, selected for secondary mutations in *fusA* and *SAUSA300_0749*. This study is the first to describe fitness compensatory events in SCVs with deletion mutations and adaptation of SCVs to continued exposure to gentamicin.

## Introduction

The long-term stability of antibiotic resistance in a bacterial population is dependent on several key parameters, namely the fitness cost of the resistance mechanism, the selection pressure for maintaining it conferred by the level of antibiotic usage in hospital and community settings and the rate of compensatory evolution ameliorating its potential biological cost (Barbosa and Levy, 2000; Andersson, 2006; Andersson and Hughes, 2010, 2011). In the majority of investigated cases, chromosomal and plasmid encoded antibiotic resistance mechanisms carry a fitness cost in terms of reduced competitiveness to sensitive isolates and are therefore selected against in the absence of antimicrobial selection pressure (Andersson, 2006; Andersson and Hughes, 2010; Vogwill and MacLean, 2015). The adverse effect of the resistance mechanism can, however, be suppressed via compensatory events, increasing fitness of the resistant organism and thereby stabilizing the resistance mechanism in the bacterial population in the absence of antimicrobial selection pressure (Andersson and Hughes, 2010). Compensatory genetic events have been extensively studied in the suppression of the adverse effects of target-site resistance mutations (Björkman et al., 1998; Reynolds, 2000; Paulander et al., 2007; Paulander et al., 2010; Brandis et al., 2012). In these cases, selection has mainly been for compensatory events restoring the enzymatic activity that was reduced by the resistance mutation (Björkman et al., 1998; Reynolds, 2000; Paulander et al., 2007, 2010; Brandis et al., 2012). Examples include rifampicin resistance mutations in *rpoB* (Reynolds, 2000; Brandis et al., 2012), streptomycin resistance mutations in *rpsL* (encoding ribosomal S12 protein) (Björkman et al., 1998) and mupirocin resistance mutations in the *ileS* (encoding isoleucyl-tRNA synthetase) (Paulander et al., 2007, 2010). Contrarily, compensation of the fitness cost conferred by non-target-site resistance mechanisms is not well understood. One example of a non-target-site resistance mechanism conferring a high fitness cost is reduced aminoglycoside (e.g., gentamicin) uptake in *S. aureus*.

Gentamicin uptake is dependent on the membrane potential, which can be greatly reduced when the flow of electrons in the electron transport chain is impaired (Taber et al., 1987). This can occur through chemical inhibition of the electron transport chain (Hoffman et al., 2006; McCollister et al., 2011) or via mutations in the pathways for menaquinone and hemin biosynthesis (Schaaff et al., 2003; von Eiff et al., 2006; Lannergård et al., 2008, 2011; Mayfield et al., 2013; Dean et al., 2014). In *S. aureus*, the electron acceptors hemin of the cytochromes and menaquinone (isoprenylated form of menadione) are required for electron flow in the electron transport chain (McNamara and Proctor, 2000). The insufficient flow of electrons in the electron transport chain that limits the establishment of the proton gradient across the cell membrane not only confers reduced uptake of aminoglycosides, but also causes large physiological changes in the cell, e.g., shifting metabolism from respiration to fermentation (McNamara and Proctor, 2000). Phenotypically the reduction in membrane potential leads to the appearance of small colonies on agar plates, hence the commonly accepted name of these being small colony variants (SCVs). In the Gram-positive opportunistic pathogen *S. aureus*, gentamicin resistant SCVs display reduced hemolysis due to decreased alpha-toxin production on blood agar plates, lack of pigmentation and often auxotrophy to either menadione or hemin (Proctor et al., 2006). The fitness cost of *S. aureus* SCVs with reduced membrane potential is associated with decreased ATP production (Proctor et al., 1998; Kohler et al., 2003), the inability to utilize of a wide range of sugars, such as mannitol, xylose, lactose, sucrose, maltose and glycerol (McNamara and Proctor, 2000) and the inability to re-utilize lactate that is produced during growth on glucose (Baumert et al., 2002). However, the SCV phenotype can be unstable in the absence of antibiotic selection pressure, which may lead to the reversion to wild type (WT) colony phenotype and the concomitant loss of aminoglycoside resistance (Lannergård et al., 2008; Tuchscherr et al., 2011).

The presences of SCVs in patients have been associated with persistent infections that are difficult to treat with antibiotic therapy, indicating a selection for this phenotype during persistent infections and antibiotic therapy (Kahl et al., 2016). Given the clinical success of SCVs and the ability to cause recurrent infections, it is important to examine compensatory adaptation to the fitness cost of SCVs and determine if concurrent antibiotic treatment affects the compensation. Previously a few studies have addressed this question, showing that reversion to normal colony phenotype can proceed via compensatory intragenic mutations in SCV isolates carrying point mutations in environments with no selection pressure (Lannergård et al., 2008; Pränting and Andersson, 2011).

The aim of the study was to use experimental evolution to determine the fitness compensatory events that take place in gentamicin resistant SCVs carrying either point mutations or deletions and assess how the presence of gentamicin affects fitness compensation.

## Materials and Methods

### Strains, Media, Auxotrophy and MIC Determination

All strains used in this study are derivatives of the *S. aureus* JE2 (**Table 1**). The following transposon mutants were retrieved from the Nebraska Transposon Mutant Library (Fey et al., 2013): JE2 *hemB*::ΦNΣ (NE1845), JE2 *menD*::ΦNΣ (NE1345), JE2 *rpoF*::ΦNΣ (NE1109) and JE2 *SAUSA300_1252*::ΦNΣ (NE142). Strains were grown in TSB at 37°C with shaking at 200 rpm or on TSA agar plates with or without addition of gentamicin (Sigma). Minimal inhibitory concentrations (MICs) of gentamicin were determined using a twofold microbroth dilution assay according to Wikler and Clinical and Laboratory Standards Institute [CLSI] (2009) guidelines, except that cation-adjusted Mueller Hinton broth was substituted with TSB. Auxotrophy of selected gentamicin resistant SCVs was determined by visual growth compensation on TSA plates supplemented with menadione (1 μg/ml) (Sigma) or hemin (1 μg/ml) (Sigma). Construction of MV118 *rpoF*::ΦNΣ (MV216) and MV118 *SAUSA300_1252*::ΦNΣ (MV218) mutants was performed by transduction with bacteriophage φ11, selecting for transductants on erythromycin plates (5 μg/ml) (Fey et al., 2013).

**Table 1:** Strains used in this study.

Strain	Description	Source
*S. aureus* JE2	CA-MRSA USA300, Erm^S^, plasmid cured LAC derivative	Fey et al., 2013
NE1845	JE2 *hemB*::ΦNΣ, Erm^R^	Fey et al., 2013
NE1345	JE2 *menD*::ΦNΣ, Erm^R^	Fey et al., 2013
NE1109	JE2 *rpoF*::ΦNΣ, Erm^R^	Fey et al., 2013
NE142	JE2 *SAUSA300_1252*::ΦNΣ, Erm^R^	Fey et al., 2013
MV108	SCV selected on gentamicin (4 μg/ml)	This study
MV112	SCV selected on gentamicin (4 μg/ml)	This study
MV118	SCV selected on gentamicin (4 μg/ml)	This study
MV123	SCV selected on gentamicin (4 μg/ml)	This study
MV127	SCV selected on gentamicin (4 μg/ml)	This study
MV216	MV118 *rpoF*::ΦNΣ, Erm^R^	This study
MV218	MV118 *SAUSA300_1252*::ΦNΣ, Erm^R^	This study

### Selection of Gentamicin Resistant SCVs

Selection of spontaneous gentamicin resistant SCVs was performed on TSA plates supplemented with 4 μg/ml of gentamicin, by applying proper dilutions of JE2 over-night (ON) cultures on the plates. SCV colonies were picked after 48 h of incubation and re-streaked on gentamicin-free TSA plates to examine the stability of the SCV phenotype. If no revertants appeared in the re-streak, one colony from each independent selection was saved. The mutation frequency of gentamicin resistant SCVs was calculated as the ratio of the number of gentamicin resistant mutants divided by the total number of cells (determined by plating proper dilutions on TSA plates).

### PCR, Sanger-Sequencing and DNA Isolation

Genomic DNA (gDNA) was isolated from 1 ml ON cell culture using DNeasy Blood and Tissue Kit (Qiagen). Cell lysis prior to gDNA isolation was achieved by pre-treating ON cultures with lysostaphin (5 mg/ml) for 1–2 h. Genes were amplified with two primer pairs to secure proper quality along the entire genes of interest. Genes of interest were amplified by PCR using Taq DNA polymerase (Thermo Scientific). Primers used for amplification are available in Supplementary Table S1. The PCR program used for amplification was: 5 min denaturation at 95°C, followed by 30 cycles of (i) denaturation 95°C for 30 s, (ii) annealing at proper temperature for 30 s for each primer pair and (iii) elongation for 1 min/kb at 72°C. A final elongation step of 5 min was included. Amplification products were verified by gel electrophoresis and DNA concentration measured on a NanoDrop 1000 (Thermo Scientific). Sanger-sequencing was performed by Macrogen Europe Inc (Amsterdam, the Netherlands).

### Genome Sequencing and Mutation Analysis

Whole genome sequencing was performed at the Department of Clinical Microbiology at Hvidovre Hospital on a MiSeq (Illumina, San Diego, CA, USA). DNA concentrations were normalized using a Qubit (Invitrogen, UK). Libraries were made with Nextera XT DNA sample preparation kit (Illumina, US), genomes multiplexed to 24 isolates per run and sequenced with 2 × 150 bp paired-end reads. Analysis of output reads from the MiSeq was performed in CLC Genomics Workbench version 8.0. Reads were aligned to the *S. aureus* USA300_FPR3757 reference genome (Genbank accession no. NC_07793). Variants were called at standard settings. To exclude false-positive variant calls due to alignment of reads to the *S. aureus* USA300_FPR3757 reference genome, we sequenced the JE2 strain that is the ancestral strain of the selected SCVs. Variants identified in our JE2 sequence were excluded in the selected SCVs and evolved strains by the function ‘Filter Variants against Control Reads.’

### Compensatory Evolution Experiment

Compensatory evolution was performed in TSB and in TSB supplemented with gentamicin at aaa12 MIC of the respective SCV strain. Five independent lineages started from single colonies for each strain were passaged under both culturing conditions. The evolution experiment was performed via serial passaging in 10 ml falcon tubes containing 1 ml growth medium in each tube for proper aeration. Every 24 h 1 μl, corresponding to approximately 10^6^ cells, was transferred into fresh growth medium, allowing for growth of approximately 10 generations per transfer. After every 50 generations of evolution, samples from each lineage were plated on TSA plates and visually inspected for growth compensation based on colony size. Restored fitness in compensated lineages could also be recognized by visual inspection of the culture cell density. If cells in a lineage reverted to normal colony phenotype, one colony was saved from that lineage. The strains not showing changes in colony size were passaged for 50 days (∼500 generations), at which point one colony from each lineage was saved.

### Fitness Measurements

Fitness was estimated by two parameters. (i) Growth rates in exponential phase were measured in a Bioscreen C reader (Oy Growth Curves Ab) at 37°C. Bacteria from ON cultures were diluted to a final concentration of 10^6^ CFU/ml in 1 ml TSB or in 1 ml TSB supplemented with gentamicin (aaa12 MIC of the parent SCV strain). Three hundred microliter was transferred to each well of the bioscreen plate with measurements taken every 5 min at 600 nm wavelength. The relative growth rates were calculated as the growth rate of the mutant divided by the growth rate of the WT strain. Three biological replicates were performed for each mutant, with three technical replicates per biological. (ii) colony forming unit (CFU) counts at stationary phase after 24 h growth was determined by growing cells in 1 ml TSB in a 10 ml falcon tube with constant shaking at 200 rpm. Cells from ON cultures were diluted to a starting inoculum of 10^6^ CFU/ml. CFU quantification was determined on TSA plates. Three biological replicates were performed for each strain.

### Membrane Potential Measurements Using Flow Cytometry

Assessment of variations in membrane potential was estimated using a flow cytometry assay based on the BacLight Bacterial Membrane Potential Kit (LifeTechnologies). Cells from ON cultures were inoculated in 30 ml TSB in 300 ml Erlenmeyer flasks and grown to an OD_600_ of 0.2. Fifteen microliter culture was transferred to 1 ml filtered phosphate-buffered saline (PBS). To each cell solution 10 μl of fluorescent membrane potential indicator dye, DiOC_2_(3), was added and cells were stained for 30 min at room temperature. Data was recorded on a BD Biosciences Accuri C6 flow cytometer (Becton, Dickinson and Company), with emission filters suitable for detecting red and green fluorescence. Settings on the flow cytometer were as follows: 50000 recorded events at a FSC threshold of 15000 and medium flow rate. Gating of stained cell population and analysis of flow cytometry data were performed in CFlow^®^ (BD Accuri). As an indicator of membrane potential the ratio of red to green fluorescence intensity was calculated. The assay was verified using the two SCV strains with transposon insertions in *hemB* (NE1845) and *menD* (NE1345).

### Statistics

Significant difference was calculated by 1-way ANOVA, with a post-hoc analysis of Tukey’s Multiple Comparison Tests (^∗^*p* < 0.05, ^∗∗^*p* < 0.01, ^∗∗∗^*p* < 0.001). Statistical analysis was performed in GraphPad Prism 4 (GraphPad Software, Inc.).

### Nucleotide Sequence Accession Numbers

Sequence reads from all isolates are deposited in the European Nucleotide Archive under study accession no. PRJEB15409.

## Results

### Mutations Conferring Resistance to Gentamicin

Spontaneous gentamicin resistant mutants were selected on TSA plates containing 4 μg/ml gentamicin, corresponding to 2x MIC of WT *S. aureus* JE2. All resistant mutants displayed a small colony phenotype and were selected with an average frequency of 2 × 10^−6^. Five mutants showing a stable small colony phenotype when restreaked on antibiotic-free plates were whole genome sequenced (WGS) in order to identify the resistance conferring mutations. Only a single mutation was identified for each mutant (**Table 2**). Four mutations were located in the menaquinone (MenA Asn198Lys and MenD Ala413fs) and hemin (HemH Glu263* and HemB Pro240Leu) biosynthesis pathways. The fifth strain contained a deletion in the *SAUSA300_1683* gene (SAUSA300_1683 Leu274fs), encoding for the bi-functional enzyme 3-deoxy-7-phosphoheptulonate synthase [EC: 2.5.1.54]/chorismate mutase [EC: 5.4.99.5]. This enzyme is required for the biosynthesis of chorismate, the first precursor molecule of the menaquinone biosynthesis pathway (Wakeman et al., 2012). All five mutants displayed a significant reduction in membrane potential, when assayed with the indicator dye, DiOC_2_(3) (**Table 2**). The reduction in membrane potential of the SCVs correlated with 8–16 times increased gentamicin MIC (16–32 μg/ml), compared to the MIC of the WT strain (2 μg/ml). The *menD* and *SAUSA300_1683* displayed menadione auxotrophy, while the *hemH* and *hemB* displayed hemin auxotrophy. Growth complementation with menadione could not be achieved for *menA* (Lannergård et al., 2011) (**Table 2**).

**Table 2:** Genotypic and phenotypic characterization of selected SCVs.

Strain	Genotype			MIC (μg/ml)	RGR	Auxotrophy	MP
	Gene	Nucleotide	AA				
JE2	WT	–	–	2	1	–	1
							
MV108	*hemH*	G787T	Glu263^∗^	32	0.64	Hemin	0.09
MV112	*SAUSA300_1683*	820_823delTTAG	Leu274fs	32	0.59	Menadione	0.07
MV118	*menD*	1237_1238delGC	Ala413fs	32	0.63	Menadione	0.09
MV123	*hemB*	C746T	Pro249Leu	16	0.60	Hemin	0.14
MV127	*menA*	T594A	Asn198Lys	32	0.72	–	0.07

*Identification of SCV conferring mutations on nucleotide and amino acid (AA) level, and phenotypic traits, such as gentamicin MIC, relative growth rate in TSB (RGR) to WT, auxotrophy to menadione and hemin, and relative impact on membrane potential (MP) assayed with DiOC_2_(3). The fluorescent dye DiOC_2_(3) exhibits green fluorescence in bacterial cells and shifts toward red fluorescence, when the dye molecules self-associate at higher cytosolic concentrations caused by larger membrane potentials. The red/green ratio of wild type WT is set to 1.*

### Fitness of Resistant Mutants

**FIGURE 1:**
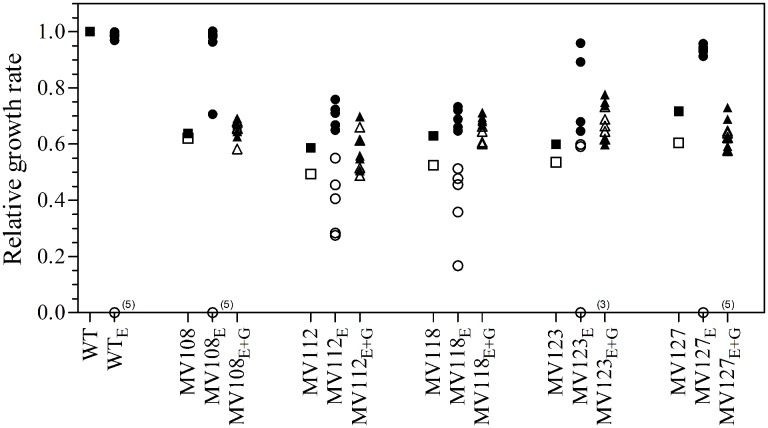
**Relative growth rates of SCV mutants and evolved lineages.** The growth rate of wild type (square) is set to 1. Relative growth rates of five ancestral SCV strains (squares), lineages evolved in TSB have the suffix E (circles) and lineages evolved in TSB containing 0.5 MIC gentamicin have the suffix E + G (triangles). Growth rates were measured in TSB (black symbols) and TSB containing 0.5 MIC gentamicin (white symbols). Several strains evolved in TSB did not grow with gentamicin at the applied concentration (number provided in parenthesis).

**FIGURE 2:**
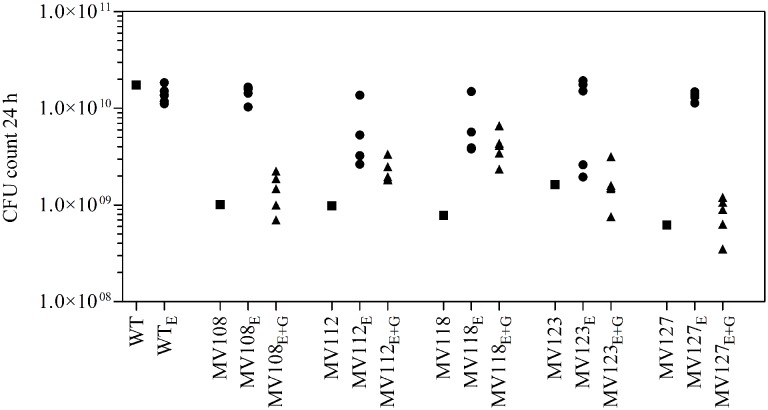
**Colony forming unit (CFU) count in stationary phase.** CFU count after 24 h growth in TSB of wild type (square), five ancestral SCV strains (squares), lineages evolved in TSB have the suffix E (circles) and lineages evolved in TSB containing gentamicin have the suffix E + G (triangles).

We estimated the fitness cost of the SCV mutations using two parameters (i) exponential growth rate and (ii) CFU in stationary phase. The relative growth rate of the five SCV mutants were 0.59–0.72 in TSB, while being 0.49–0.62 in TSB supplemented with gentamicin compared to the WT strain grown in TSB (**Table 2**; **Figure 1**). Furthermore, the SCVs experienced 10–30 fold lower CFUs in stationary phase, when grown in TSB (**Figure 2**).

### Fitness Compensation of SCV Mutants

To investigate if fitness could be ameliorated in the SCVs and address the compensatory mechanisms involved, we conducted an evolution experiment with and without sub-lethal concentrations of gentamicin (aaa12 MIC), selecting for mutants with improved fitness. For each SCV mutant we passaged five lineages without and five lineages with gentamicin and at regular intervals screened for higher fitness by visual examination of bacterial colony size on TSA plates. To separate potential adaptation to the growth medium from SCV specific compensatory events we passaged five WT lineages for 500 generations in TSB in parallel. None of the evolved WT lineages displayed improved fitness in terms of exponential growth rate or stationary CFU count, indicating that *S. aureus* JE2 is generally well adapted to growth in TSB (**Figures 1** and **2**).

**FIGURE 3:**
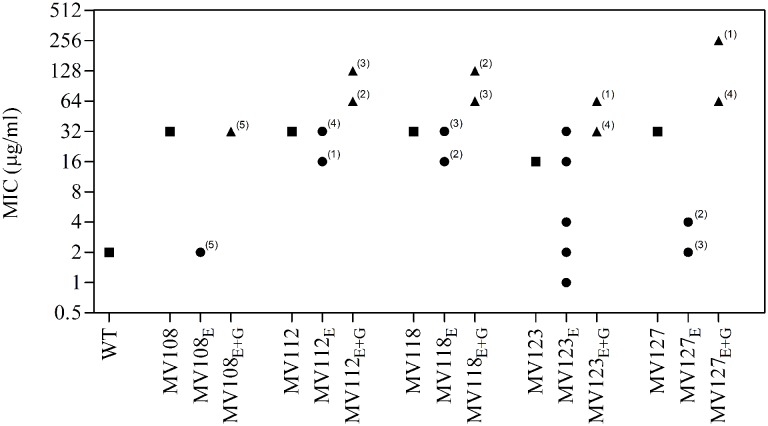
**Gentamicin minimum inhibitory concentration of SCV mutants and evolved lineages.** Gentamicin MIC of wild type (square), five ancestral SCV strains (squares), lineages evolved in TSB have the suffix E (circles) and lineages evolved in TSB containing gentamicin have the suffix E + G (triangles). Numbers indicate number of lineages with the respective MIC.

Out of the 50 passaged lineages, only 12 reverted to WT colony size (Supplementary Table S2). All of the 12 lineages originated from SCVs containing point mutations (*hemH*, *menA* or *hemB*) and the reversion occurred only when passaged without gentamicin. Interestingly, in a single passage (∼10 generations) revertants with WT colony size accounted approximately for 50% of the total colonies in the population (tested upon plating on antibiotic-free plates), where no revertants had been observed at the former passage. This fast take over cannot be explain solely with increased growth rates but might also indicate that revertants utilize nutrients that the SCV mutant cannot, which is supported by the observation that revertants grow to approximately 10-fold higher cell densities than SCVs (**Figure 2**). Reversion to WT colony size proceeded within 50–310 generations for the 12 lineages. As the reverted clones in a single passage constituted 50% of the population, the rate for reversion to WT colony phenotype was treated as a stochastic event. The rate of reversion was estimated to be approximately 10^−8^–10^−9^ per cell per generation (Maisnier-Patin et al., 2002), similar to previous reversion rates of SCV mutants with point mutations (Lannergård et al., 2008; Pränting and Andersson, 2011). Fitness in terms of exponential growth rate and stationary CFU of the 12 lineages increased to WT levels (**Figures 1** and **2**). The relative growth rates for the compensated strains ranged from 0.89–1.00, compared to 0.60–0.72 of the ancestral SCVs (**Figure 1**). The lineages also gained sensitivity toward gentamicin (**Figure 3**) and an assessment of fitness was therefore not possible to do in TSB supplemented with gentamicin (**Figure 1**).

None of the 10 lineages derived from deletion SCV mutants (*menD* and *SAUSA300_1683*) and evolved without gentamicin reverted to WT colony phenotype, even though they displayed improved fitness in terms of growth rate and CFU in stationary phase (**Figures 1** and **2**). The relative growth rates in TSB of *menD* lineages were 0.65–0.73 compared to 0.63 of the ancestral *menD*, while the relative growth rates of *SAUSA300_1683* lineages were 0.65–0.76 compared to 0.59 of the ancestral *SAUSA300_1683*. Only three of the lineages displayed twofold reduced MIC compared to respective ancestral SCVs, while the remaining seven lineages retained the same MIC as respective ancestral SCVs (**Figure 3**). The increased growth rates of the 10 lineages resulted in a trade-off with significantly reduced growth rates for the majority of the lineages, when grown in TSB supplemented with gentamicin (**Figure 1**).

None of the 25 lineages evolved with gentamicin restored colony phenotype to WT levels. Nonetheless, the majority of lineages (*n* = 22/25) displayed improved growth rates in TSB supplemented with gentamicin, while the growth rates were increased for 15/25 in TSB (**Figure 1**). Increased levels of resistance to gentamicin (2–8 fold) were observed for 20/25 lineages (**Figure 3**).

### Fitness Compensation via Restoration of Membrane Potential

**FIGURE 4:**
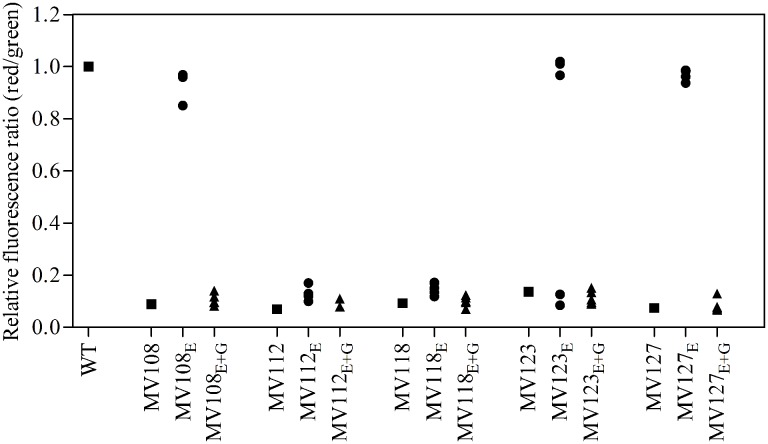
**Assay for membrane potential of SCV mutants and evolved lineages.** The membrane potential was assayed using the fluorescent dye DiOC_2_(3), which exhibits green fluorescence in bacterial cells and shifts toward red fluorescence, when the dye molecules self-associate at higher cytosolic concentrations caused by larger membrane potentials. The red/green ratio of wild type (square), five ancestral SCV strains (squares), lineages evolved in TSB have the suffix E (circles) and lineages evolved in TSB containing gentamicin have the suffix E + G (triangles).

For the point mutation SCVs evolved in TSB, Sanger-sequencing of the respective SCV conferring genes (*hemH*, *menA* or *hemB*) revealed intracodonic mutations in 12/15 lineages (**Table 3**). We identified reversion to WT sequence on nucleotide level in eight lineages and for two lineages on amino acid level. In two lineages, compensatory mutations substituted the SCV conferring amino acid with a non-synonymous amino acid compared to the WT sequence. The acquisition of intracodonic compensatory mutations in the 12 lineages correlated with reversion of membrane potential to the level of the WT (**Figure 4**) and gentamicin re-sensitization (**Figure 3**). In the remaining three lineages, we did not identify any intragenic mutations in the SCV conferring genes. These lineages indeed displayed lower levels of fitness amelioration compared to the lineages carrying intracodonic compensatory mutations. The two lineages (E68 and E69) retained membrane potentials as ancestral SCV and remained resistant to gentamicin, while lineage E47 displayed partial growth rate and membrane potential restoration and gentamicin re-sensitization (Supplementary Table S2).

**Table 3:** Spectrum of intragenic compensatory mutations in evolved lineages derived from SCVs with point mutations.

			Compensatory mutation	
Strain	Lineage	SCV mutation	Nucleotide	Amino acid
MV108		*hemH* G787T	–	–
	E1	*hemH* G787T	T787G	^∗^263Glu
	E4	*hemH* G787T	T787G	^∗^263Glu
	E21	*hemH* G787T	T787G	^∗^263Glu
	E24	*hemH* G787T	T787G	^∗^263Glu
	E47	*hemH* G787T	–	–
MV123		*hemB* C746T	–	–
	E35	*hemB* C746T	T746C	Leu249Pro
	E41	*hemB* C746T	T746C	Leu249Pro
	E50	*hemB* C746T	T746C	Leu249Pro
	E68	*hemB* C746T	–	–
	E69	*hemB* C746T	–	–
MV127		*menA* T594A	–	–
	E7	*menA* T594A	A592G	Lys198Glu
	E15	*menA* T594A	A594T	Lys198Asn
	E18	*menA* T594A	A593C	Lys198Thr
	E38	*menA* T594A	A594C	Lys198Asn
	E44	*menA* T594A	A594C	Lys198Asn

### Inactivation of σ^B^ Stress Response Improves Fitness of SCVs in Absence of Gentamicin

**FIGURE 5:**
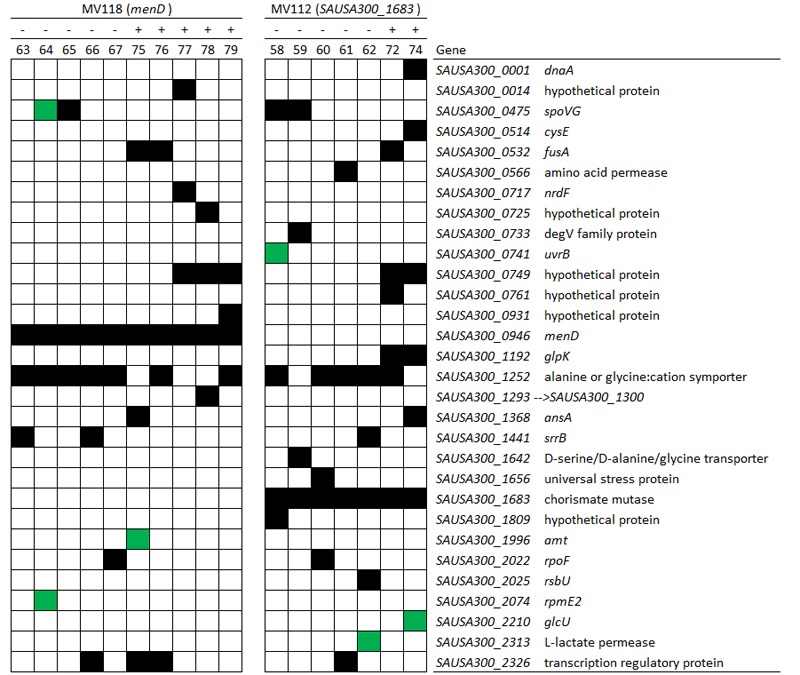
**Spectrum of compensatory mutations identified in evolved lineages.** Mutations in genes identified in evolved lineages derived from deletion SCVs (*menD* and *SAUSA300_1683*) based on whole genome sequencing. The black squares in the matrix denote that the gene contains a mutation. Green squares in the matrix denote a mutation within 250 bp proximally of the start codon. (-) indicate lineages evolved without gentamicin and (+) indicate lineages evolved with gentamicin. For detailed information about the mutations, see Supplementary Table S3.

To identify compensatory mechanisms in the lineages that did not ameliorate the fitness cost via restoration of the membrane potential, we WGS all lineages derived from deletion SCV mutants (*menD* and *SAUSA300_1683*) exhibiting improved fitness, as well as the five WT control lineages. All of the evolved SCV lineages still contained the original SCV mutation (**Figure 5**; Supplementary Table S3). We identified multiple genes that acquired mutations in parallel lineages, suggesting selection for such changes. No mutations from the control lineages were identified in the evolved SCV lineages, suggesting that mutations in the SCV lineages were specific to the effect of the SCV mutations and not due to general adaptation to the growth environment.

**FIGURE 6:**
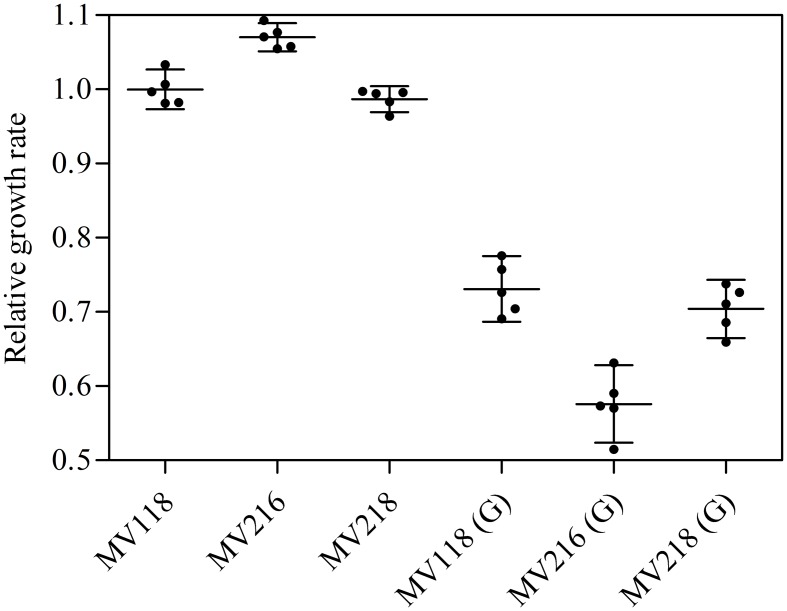
**σ^B^-mediated fitness cost amelioration and associated trade-off.** The relative growth rates of the SCV strain (MV118) and the derivatives MV216 (MV118 *rpoF*::ΦNΣ) and MV218 (MV118 *SAUSA300_1252*::ΦNΣ) measured in the absence and presence of gentamicin (G).

From the *menD* and *SAUSA300_1683* lineages evolved without gentamicin, 7/10 acquired mutations in genes related to the σ^B^ stress response [*spoVG* (*n* = 4), *rsbU* (*n* = 1) and *rpoF* (*n* = 2)]. The *spoVG* gene encodes for the downstream modulator SpoVG of the σ^B^ general stress response (Schulthess et al., 2011), *rsbU* encodes for the RsbU activator of σ^B^ (Hecker et al., 2007), while *rpoF* encodes for the alternative sigma factor SigB (σ^B^) (Hecker et al., 2007). None of the seven sequenced lineages evolved with gentamicin had acquired mutations related to the σ^B^ stress response. To determine whether inactivation of the σ^B^ response confers a fitness advantage in TSB we inactivated *rpoF* in MV118 with a transposon insertion derived from the Nebraska Transposon Mutant Library (Fey et al., 2013). The exponential growth rate of this mutant (MV216) was 7% higher in TSB than ancestral MV118, however, displayed a significant growth defect in TSB supplemented with gentamicin (**Figure 6**). This correlates well with the observed growth rate trade-off in the majority of *menD* and *SAUSA300_1683* lineages evolved without gentamicin (**Figure 1**). Furthermore, three of the lineages evolved without gentamicin displayed non-synonymous missense mutations in the sensor histidine kinase *srrB* of the staphylococcal respiratory response two-component system SrrAB (Kinkel et al., 2013). Two of these lineages did not contain any σ^B^-related mutations.

Independent of whether *menD* and *SAUSA300_1683* lineages evolved with or without gentamicin, 12/17 lineages acquired mutations in the gene *SAUSA300_1252*, encoding a alanine/glycine:cation symporter. Premature stop-codons and INDELs causing frameshifts suggest that the symporter is non-essential and that the mutations cause a loss of function. We inactivated *SAUSA300_1252* with a transposon insertion in SCV strain MV118 (MV218) to assay the fitness effect in TSB and TSB supplemented with gentamicin. We could not identify any difference in terms of exponential growth rate between MV118 and MV218 in TSB or TSB supplemented with gentamicin (**Figure 6**).

### Evolution in the Presence of Gentamicin Selects for Additional Resistance Mutations

Increased levels of gentamicin resistance were detected in 20/25 of the lineages evolved with gentamicin compared to respective ancestral SCV mutants (**Figure 4**). We WGS seven *menD* and *SAUSA300_1683* lineages evolved in the presence of gentamicin. All of the seven lineages contained mutations in either *fusA* and/or *SAUSA300_0749*, while none of the lineages evolved without gentamicin contained mutations in these genes (**Figure 5**). The gene *fusA* encodes the elongation factor G (EF-G) and mutations were identified in three lineages (E72, E75, and E76). The combination of *fusA* and a SCV conferring mutations has previously been described for *in vitro* selected kanamycin resistant mutants in *S. aureus* (Norström et al., 2007; Lannergård et al., 2011). The gene *SAUSA300_0749* encodes a product of unknown function and has not previously been associated with gentamicin resistance. A premature stop-codon in strain E74 (Gln3^∗^) suggests that the gene is non-essential and that loss-of-function confer the resistance.

## Discussion

The impact of antibiotic resistance and compensatory events on an organism’s fitness are important parameters to consider when assessing the evolution of antibiotic resistance and the risk of its persistence in bacterial populations (Andersson and Hughes, 2010, 2011; Hughes and Andersson, 2015). Experimental evolution has been widely used to assess these two parameters and the genetic mechanisms involved in reducing the fitness cost associated with antibiotic resistance. These studies have mainly been focused on the fitness cost associated with target-site resistance mechanisms and how this cost can be compensated for (Björkman et al., 1998, 2000; Paulander et al., 2007, 2010; Brandis et al., 2012). Therefore, we wanted to investigate how the fitness cost of the non-target-site aminoglycoside resistance mechanism conferred by membrane de-polarization, may be compensated for in the clinically relevant bacterial pathogen *S. aureus.*

The largest fitness increase was observed in lineages derived from SCVs with a point mutation (*menA*, *hemB* and *hemH*) that evolved without gentamicin. In 12/15 lineages, evolved mutants displayed WT colony morphology and relative fitness of 0.89–1.00 compared to 0.60–0.72 of the ancestral SCVs (**Figure 1**). The fitness compensation correlated with restoration of membrane potential (**Figure 4**) and re-sensitization to gentamicin (**Figure 3**). Sequencing of the respective resistance genes revealed intracodonic suppressor mutations in all of the 12 lineages, leading to either nucleotide reversion to WT sequence, reversion to WT sequence on amino acid level or substitution of the SCV conferring amino acid mutation (**Table 3**). For the remaining three lineages that did not display fully WT phenotype characteristics, we could not identify any secondary intragenic mutations. Restored fitness has previously been associated with reversion in *S. aureus* SCV isolates with point mutations as the resistance conferring mutation (Lannergård et al., 2008; Dean et al., 2014). For target-site resistance mechanisms, compensation via reversion is rarely selected for (Levin et al., 2000; Paulander et al., 2007; Andersson and Hughes, 2010; Brandis et al., 2012). This is a consequence of the higher frequency of compensatory events (intragenic, extragenic and amplifiaction) restoring fitness compared to the frequency of reversion mutations and the bottlenecks associated with serial passage, leading to higher probability of the loss of reversion mutations due to genetic drift (Levin et al., 2000). We hypothesize that revertants of SCV isolates are not lost due to genetic drift in our experimental setup, as within a single passage revertants constituted approximately 50% of the population and cell density increased 10-fold (**Figure 2**). Rather this indicates that revertants can utilize nutrients that SCVs cannot.

Selection for restoration of membrane potential to WT level only occurred in lineages derived from point mutation SCVs that evolved without gentamicin. In the evolved lineages that originated from SCVs with deletions, restoration of membrane potential did not occur under any growth conditions. The relative growth rate increased 2–17% (**Figure 1**) in these lineages, but the parental SCV level of gentamicin resistance was maintained (**Figure 3**). For 7/10 of lineages derived from SCVs with deletions evolved without gentamicin, we detected mutations related to the σ^B^ stress response, namely [*rpoF* (2/10), *rsbU* (1/10) and *spoVG* (4/10) lineages]. Furthermore, we detected mutations in alanine/glycine transporter encoded by the gene *SAUSA300_1252* in 9/10 lineages (**Figure 5**). The introduction of pre-mature stop-codons or frame-shift mutations indicated that the mutations caused a loss-of-function of the gene products. We could show that transposon inactivation of *rpoF* in a SCV mutant conferred improved exponential growth rates in the absence of gentamicin, but lower growth rates with gentamicin, correlating with the observation of the passaged lineages containing mutations in σ^B^ stress related genes (**Figure 6**).

The σ^B^ stress response regulates the expression of multiple non-specific stress mechanisms and virulence factors in *S. aureus* (Chan et al., 1998). In a SCV isolate with a *hemB* mutation it has been established that the σ^B^ stress response is permanently activated, although the signal transduction behind this activation remains unknown (Senn et al., 2005; Mitchell et al., 2013). Decreased levels of ATP, as observed in ETC-deficient SCVs (Kohler et al., 2003), seem not to be an activator of the σ^B^ stress response in *S. aureus* (Pané-Farré et al., 2006), as *S. aureus* lacks the energy sensor RsbP of *Bacillus subtilis* (Hecker et al., 2007). A functional σ^B^ stress response, however, is important in the initial selection of gentamicin resistant SCVs (Mitchell et al., 2010a,b), but our data indicates that a continuously activated σ^B^ in SCVs impose a fitness cost and is selected against *in vitro* in the absence of antibiotic selection. Though we describe a significant fitness cost associated with the σ^B^ stress response in SCVs *in vitro*, a functional σ^B^ stress response has been reported as an important mediator in the switching from acute infection to a silent SCV-phenotype leading to intracellular persistence (Tuchscherr et al., 2015).

To assess compensatory evolution in the presence of gentamicin, we selected for mutants with increased fitness at sub-lethal (1/2 MIC) concentrations of gentamicin. At this concentration, the exponential growth rates were further decreased for the ancestral SCVs compared to growth in antibiotic-free medium, suggesting that membrane-depolarization does not completely prevent uptake of gentamicin (**Figure 1**). The SCV lineages evolved in presence of gentamicin (1/2 MIC) experienced enhanced resistance to gentamicin (20/25 lineages) (**Figure 3**) and increased growth rates in the presence of gentamicin (22/25 lineages) (**Figure 1**). Missense mutations in *fusA* (3/7 lineages) and/or mutations in *SAUSA300_0749* (5/7 lineages) most likely accounted for the increased gentamicin MIC, as they were identified in multiple parallel lineages and exclusively in lineages passaged with gentamicin (**Figure 5**). Mutations in *fusA* that encodes for the ribosomal translation elongation factor, EF-G, can confer resistance to fusidic acid and aminoglycosides and *fusA* mutations in SCV strains have previously been observed (Lannergård et al., 2011). The gene product of *SAUSA300_0749* is a hypothetical protein of unknown function and has not previously been described in relation to gentamicin resistance.

Our data show that accumulation of only few *de novo* mutations confer high-level resistance to aminoglycosides, which can be of clinical importance. Selection for increased fitness in the presence of antibiotics via serial passage has also been described for target-site resistance mechanisms, such as rifampicin resistant *rpoB* mutants (Brandis et al., 2012) and mupirocin resistant *ileS* mutants (Paulander et al., 2007). Here the majority of the compensatory events related to acquisition of additional intragenic mutations, gene amplifications or suppressor mutations in related genes (Paulander et al., 2007; Brandis et al., 2012), e.g., mutations in other ribosomal genes (*rpoA* and *rpoC*) in an *rpoB* mutant (Brandis et al., 2012). The data suggests that where target-site mutations may increase the activity of a target enzyme, while still displaying reduced affinity of the antibiotic, then further reductions of membrane potential do not seem feasible for SCVs and therefore non-membrane potential resistance mechanisms are selected for.

## Conclusion

Our study highlights three compensatory pathways to ameliorate the fitness cost associated with SCV gentamicin resistance. The trajectories of fitness compensation depended on initial mutation type (point mutation vs. deletion), and whether evolution proceeded with or without gentamicin. Switching between fast growing variants and slow growing of *S. aureus* is associated with clinically persistent and recurrent infections (Proctor et al., 2006). Membrane de-polarization and membrane potential restoration as demonstrated here provides such a switching mechanism. Whole genome sequencing of clinical SCV isolates in long-term infections could reveal whether SCVs with mutations in σ^B^ stress response genes is also selected for in the human host or whether this compensatory event is restricted to laboratory conditions. Finally, we demonstrate that continued gentamicin selection pressure can select for additional resistance mutations increasing gentamicin resistance. Contrarily, gentamicin selection pressure could potentially be used to trap SCVs in a low fitness state to limit recurrence of acute infection and then provide a treatment opportunity with compounds specifically targeting SCVs (Mitchell et al., 2011).

## Author Contributions

MV, WP, and HI conceived and designed the study. Experiments were performed by MV, WP, BL, and JN. MV, WP, JN, HW, and HI analyzed the data. MV, WP, HW, and HI drafted the manuscript. All authors read and approved the final manuscript.

## Conflict of Interest Statement

The authors declare that the research was conducted in the absence of any commercial or financial relationships that could be construed as a potential conflict of interest.
