# Supplementary material for: Novel Pathways for Ameliorating the Fitness Cost of Gentamicin Resistant Small Colony Variants
Source: Front Microbiol. 2016 Nov 22;7:1866. doi: 10.3389/fmicb.2016.01866 (PMC5119051; doi:10.3389/fmicb.2016.01866)
Supplement: Supplementary file 1 [file Table_1.DOCX]

**Supplementary Table 1**

Primers used for sequencing of the respective genes.

| Gene | Primer name | Sequence |
| --- | --- | --- |
| *hemB* | *hemB_F1* | CAACCAGTAACAATTGCAGAAA |
|  | *hemB_R1* | TCATAAAGTAAATTCCTCCTACAGTTT |
|  | *hemB_F2* | GTCATTGTGGCGTGATTGAT |
|  | *hemB_R2* | TCAGCAACCTTCATTGCTTC |
|  |  |  |
| *menA* | *menA _F1* | TTATCTTGCAATTTTTCACGTC |
|  | *menA _R1* | GATGTAAGTCGATTGCCGAGT |
|  | *menA_F2* | GCCCTTTCCCTATTTCATGG |
|  | *menA_R2* | AAAAACTCGACAGTTCGATGTAAG |
|  |  |  |
| *hemH* | *hemH _F1* | AAGTAAGCGAATTCGTACACACA |
|  | *hemH _R1* | ACCTGTTATCCCCGCTCCT |
|  | *hemH _F2* | GCTGATGAAGAAGCTGCAAA |
|  | *hemH _R2* | CCTGTTATCCCCGCTCCTAT |
