# Supplementary material for: Novel Pathways for Ameliorating the Fitness Cost of Gentamicin Resistant Small Colony Variants
Source: Front Microbiol. 2016 Nov 22;7:1866. doi: 10.3389/fmicb.2016.01866 (PMC5119051; doi:10.3389/fmicb.2016.01866)
Supplement: Supplementary file 2 [file Table_2.DOCX]

**Supplementary Table 2**

Characterization of fitness compensated clones upon evolution in the absence (-) or presence (+) of gentamicin, generations evolved, colony morphology (WT or SCV), the relative growth rate (RGR) in absence (-G) or presence of gentamicin (+G), MIC to gentamicin (µg/ml) and relative impact on membrane potential when assayed with DiOC_2_(3). n.d. (Not determined).

| Strain number | Strain designation | Evolution  (-/+ G) | Generations evolved | Colony morph. | RGR  (-G) | RGR  (+G) | MIC  (µg/ml) | MP |
| --- | --- | --- | --- | --- | --- | --- | --- | --- |
| MV1 | WT |  |  | WT | 1 | n.d. | 2 | 1 |
|  | E53 | - | 500 | WT | 0.97 | n.d. | n.d. | n.d. |
|  | E54 | - | 500 | WT | 0.99 | n.d. | n.d. | n.d. |
|  | E55 | - | 500 | WT | 0.98 | n.d. | n.d. | n.d. |
|  | E56 | - | 500 | WT | 1.00 | n.d. | n.d. | n.d. |
|  | E57 | - | 500 | WT | 0.98 | n.d. | n.d. | n.d. |
|  |  |  |  |  |  |  |  |  |
| MV108 | *hemH* |  |  | SCV | 0.64 | 0.62 | 32 | 0.09 |
|  | E21 | - | 140 | WT | 0.99 | n.d. | 2 | 0.96 |
|  | E47 | - | 310 | WT-SCV | 0.71 | n.d. | 2 | 0.85 |
|  | E1 | - | 50 | WT | 0.96 | n.d. | 2 | 0.97 |
|  | E4 | - | 100 | WT | 0.98 | n.d. | 2 | 0.97 |
|  | E24 | - | 140 | WT | 1.00 | n.d. | 2 | 0.96 |
|  | E90 | + | 500 | SCV | 0.63 | 0.58 | 32 | 0.08 |
|  | E91 | + | 500 | SCV | 0.67 | 0.68 | 32 | 0.10 |
|  | E92 | + | 500 | SCV | 0.64 | 0.65 | 32 | 0.12 |
|  | E93 | + | 500 | SCV | 0.67 | 0.68 | 32 | 0.10 |
|  | E94 | + | 500 | SCV | 0.69 | 0.66 | 32 | 0.14 |
|  |  |  |  |  |  |  |  |  |
| MV112 | *SAUSA1683* |  |  | SCV | 0.59 | 0.49 | 32 | 0.07 |
|  | E58 | - | 500 | SCV | 0.76 | 0.46 | 32 | 0.10 |
|  | E59 | - | 500 | SCV | 0.71 | 0.28 | 32 | 0.13 |
|  | E60 | - | 500 | SCV | 0.67 | 0.41 | 32 | 0.17 |
|  | E61 | - | 500 | SCV | 0.72 | 0.55 | 32 | 0.17 |
|  | E62 | - | 500 | SCV | 0.65 | 0.28 | 16 | 0.12 |
|  | E70 | + | 500 | SCV | 0.56 | 0.52 | 128 | 0.11 |
|  | E71 | + | 500 | SCV | 0.55 | 0.51 | 64 | 0.08 |
|  | E72 | + | 500 | SCV | 0.61 | 0.61 | 128 | 0.11 |
|  | E73 | + | 500 | SCV | 0.51 | 0.49 | 128 | 0.08 |
|  | E74 | + | 500 | SCV | 0.70 | 0.66 | 64 | 0.08 |
|  |  |  |  |  |  |  |  |  |
| MV118 | *menD* |  |  | SCV | 0.63 | 0.53 | 32 | 0.09 |
|  | E63 | - | 500 | SCV | 0.66 | 0.17 | 16 | 0.17 |
|  | E64 | - | 500 | SCV | 0.72 | 0.36 | 32 | 0.17 |
|  | E65 | - | 500 | SCV | 0.73 | 0.51 | 32 | 0.15 |
|  | E66 | - | 500 | SCV | 0.69 | 0.46 | 16 | 0.13 |
|  | E67 | - | 500 | SCV | 0.65 | 0.48 | 32 | 0.12 |
|  | E75 | + | 500 | SCV | 0.67 | 0.61 | 128 | 0.10 |
|  | E76 | + | 500 | SCV | 0.71 | 0.60 | 128 | 0.11 |
|  | E77 | + | 500 | SCV | 0.69 | 0.61 | 64 | 0.07 |
|  | E78 | + | 500 | SCV | 0.68 | 0.64 | 64 | 0.10 |
|  | E79 | + | 500 | SCV | 0.69 | 0.66 | 64 | 0.12 |
|  |  |  |  |  |  |  |  |  |
| MV123 | *hemB* |  |  | SCV | 0.60 | 0.54 | 16 | 0.14 |
|  | E68 | - | 500 | SCV | 0.68 | 0.59 | 16 | 0.08 |
|  | E69 | - | 500 | SCV | 0.65 | 0.60 | 32 | 0.13 |
|  | E35 | - | 160 | WT | 0.96 | n.d. | 4 | 1.02 |
|  | E50 | - | 310 | WT | 0.89 | n.d. | 1 | 0.97 |
|  | E41 | - | 230 | WT | 0.96 | n.d. | 2 | 1.01 |
|  | E95 | + | 500 | SCV | 0.62 | 0.73 | 32 | 0.10 |
|  | E96 | + | 500 | SCV | 0.60 | 0.66 | 32 | 0.13 |
|  | E97 | + | 500 | SCV | 0.74 | 0.69 | 64 | 0.09 |
|  | E98 | + | 500 | SCV | 0.78 | 0.62 | 32 | 0.15 |
|  | E99 | + | 500 | SCV | 0.75 | 0.64 | 32 | 0.11 |
|  |  |  |  |  |  |  |  |  |
| MV127 | *menA* |  |  | SCV | 0.72 | 0.60 | 32 | 0.07 |
|  | E38 | - | 190 | WT | 0.96 | n.d. | 2 | 0.99 |
|  | E44 | - | 300 | WT | 0.94 | n.d. | 2 | 0.98 |
|  | E15 | - | 140 | WT | 0.93 | n.d. | 2 | 0.94 |
|  | E18 | - | 140 | WT | 0.93 | n.d. | 4 | 0.98 |
|  | E7 | - | 90 | WT | 0.91 | n.d. | 4 | 0.96 |
|  | E80 | + | 500 | SCV | 0.69 | 0.64 | 256 | 0.08 |
|  | E81 | + | 500 | SCV | 0.63 | 0.62 | 64 | 0.13 |
|  | E82 | + | 500 | SCV | 0.73 | 0.58 | 64 | 0.08 |
|  | E83 | + | 500 | SCV | 0.58 | 0.62 | 64 | 0.07 |
|  | E84 | + | 500 | SCV | 0.59 | 0.65 | 64 | 0.07 |
