# Supplementary material for: Novel Pathways for Ameliorating the Fitness Cost of Gentamicin Resistant Small Colony Variants
Source: Front Microbiol. 2016 Nov 22;7:1866. doi: 10.3389/fmicb.2016.01866 (PMC5119051; doi:10.3389/fmicb.2016.01866)
Supplement: Supplementary file 3 [file Table_3.DOCX]

**Supplementary Table 3**

Mutations identified in the evolved lineages compared to WT.

|  |  |  | Mutation | |
| --- | --- | --- | --- | --- |
| Strain |  | Gene | Nucleotide | AA |
| ***menD* strains** |  |  |  |  |
| E63 | 1 | *menD* | 1237_1238delGC | Ala413fs |
|  | 2 | *SAUSA300_1252* | G791A | Gly264Asp |
|  | 3 | *srrB* | G1276C | Ala426Pro |
|  |  |  |  |  |
| E64 | 1 | *spoVG* | 10bp upstream spoVG G532990T | - |
|  | 2 | *menD* | 1237_1238delGC | Ala413fs |
|  | 3 | *SAUSA300_1252* | C382T | Gln128* |
|  | 4 | *-* | 57bp upstream rpmE2 ribosomal protein L31 C2235991T | - |
|  |  |  |  |  |
| E65 | 1 | *spoVG* | C188T | Pro63Leu |
|  | 2 | *menD* | 1237_1238delGC | Ala413fs |
|  | 3 | *SAUSA300_1252* | G289A | Ala97Thr |
|  |  |  |  |  |
| E66 | 1 | *menD* | 1237_1238delGC | Ala413fs |
|  | 2 | *SAUSA300_1252* | 347_356delGTGCAGCGAG | Gly116fs |
|  | 3 | *srrB* | C644A | Ala215Asp |
|  | 4 | *SAUSA300_1792* | C1293T | Synonymous |
|  | 5 | *SAUSA300_2326* | G889T | Asp297Tyr |
|  |  |  |  |  |
| E67 | 1 | *menD* | 1237_1238delGC | Ala413fs |
|  | 2 | *SAUSA300_1252* | C878T | Ala293Val |
|  | 3 | *rpoF* | A733C | Thr245Pro |
|  |  |  |  |  |
| E75 | 1 | *SAUSA300_0414* | C114T | Synonymous |
|  | 2 | *fusA* | G1438T | Val480Phe |
|  | 3 | *menD* | 1237_1238delGC | Ala413fs |
|  | 4 | *ansA* | A377T | Asn126Ile |
|  | 5 | *-* | 14bp upstream atm delT |  |
|  | 6 | *SAUSA300_2326* | A952T | Ile318Phe |
|  |  |  |  |  |
| E76 | 1 | *SAUSA300_0414* | C114T | Synonymous |
|  | 2 | *fusA* | G1438T | Val480Phe |
|  | 3 | *menD* | 1237_1238delGC | Ala413fs |
|  | 4 | *SAUSA300_1252* | G1073T | Gly358Val |
|  | 5 | *SAUSA300_2326* | A952T | Ile318Phe |
|  |  |  |  |  |
| E77 | 1 | *SAUSA300_0014* | C1409G | Thr470Arg |
|  | 2 | *nrdF* | G176A | Gly59Asp |
|  | 3 | *SAUSA300_0749* | 574delA | Ile192fs |
|  | 4 | *menD* | 1237_1238delGC | Ala413fs |
|  |  |  |  |  |
| E78 | 1 | *SAUSA300_0725* | T298C | Ser100Pro |
|  | 2 | *SAUSA300_0749* | A121G | Ser41Gly |
|  | 3 | *menD* | 1237_1238delGC | Ala413fs |
|  | 4 | *LysA, SAUSA300_1294 🡪 SAUSA300_1299, brnQ* | 1424713_1430350del5638bp |  |
|  |  |  |  |  |
| E79 | 1 | *SAUSA300_0749* | C482T | Pro161Leu |
|  | 2 | *SAUSA300_0931* | 134insA | Glu45fs |
|  | 3 | *menD* | 1237_1238delGC | Ala413fs |
|  | 4 | *SAUSA300_1252* | 534delT | Asn178fs |
|  |  |  |  |  |
| **SAUSA300_1683 strains** | | |  |  |
| E58 | 1 | *SAUSA300_0475* | C103T | Arg35Cys |
|  | 2 |  | 95 bp upstream uvrB C823849T |  |
|  | 3 | *SAUSA300_1252* | C226T | Gln76* |
|  | 4 | *SAUSA300_1683* | 820_823delTTAG | Leu274fs |
|  | 5 | *SAUSA300_1809* | C875T | Thr292Ile |
|  |  |  |  |  |
| E59 | 1 | *SAUSA300_0475* | C177A | Asp59Glu |
|  | 2 | *SAUSA300_0733* | 505_506insA | Gln169fs |
|  | 3 | *SAUSA300_1642* | C119T | Pro40Leu |
|  | 4 | *SAUSA300_1683* | 820_823delTTAG | Leu274fs |
|  |  |  |  |  |
| E60 | 1 | *SAUSA300_1252* | 897_910delCCCTGTTAAGCAAG | His299fs |
|  | 2 | *SAUSA300_1656* | C224T | Ala75Val |
|  | 3 | *SAUSA300_1683* | 820_823delTTAG | Leu274fs |
|  | 4 | *rpoF* | 50delA | Asn17fs |
|  |  |  |  |  |
| E61 | 1 | *SAUSA300_0566* | G840C | Synonymous |
|  | 2 | *SAUSA300_1252* | C536T | Thr179Ile |
|  | 3 | *SAUSA300_1683* | 820_823delTTAG | Leu274fs |
|  | 4 | *SAUSA300_2326* | T953C | Ile318Thr |
|  |  |  |  |  |
| E62 | 1 | *SAUSA300_1252* | 575_576insAGTAATCACAGGT | Pro192fs |
|  | 2 | *srrB* | G1040A | Gly347Asp |
|  | 3 | *SAUSA300_1683* | 820_823delTTAG | Leu274fs |
|  | 4 | *rsbU* | G365T | Gly122Val |
|  | 5 | *-* | 133 bp upstream of SAUSA300_2313 L-lactate permease (T2487644C) |  |
|  |  |  |  |  |
| E72 | 1 | *fusA* | C1432T | Pro478Ser |
|  | 2 | *SAUSA300_0749* | 422delT | Val141fs |
|  | 3 | *SAUSA300_0761* | C171A | Tyr57* |
|  | 4 | *glpK* | C788T | Thr263Ile |
|  | 5 | *SAUSA300_1683* | 820_823delTTAG | Leu274fs |
|  | 6 | *SAUSA300_1252* | 548_557del |  |
|  |  |  |  |  |
| E74 | 1 | *dnaA* | G772C | Glu258Lys |
|  | 2 | *cysE* | T485C | Val162Ala |
|  | 3 | *SAUSA300_0749* | C7T | Gln3* |
|  | 4 | *glpK* | C118T | Gln40* |
|  | 5 | *ansA* | 866delT | Ile289fs |
|  | 6 | *SAUSA300_1683* | 820_823delTTAG | Leu274fs |
|  | 7 | *-* | 133 bp upstream of glcU |  |
|  |  |  |  |  |
| Control strains |  |  |  |  |
| 1 | 1 | *purR* | T686G | Val229Gly |
| 2 | 1 | *purR* | T686G | Val229Gly |
| 3 | 1 | *purR* | T686G | Val229Gly |
| 4 | 1 |  | 163 bp upstream of *purE* G1059346T |  |
| 5 | 1 | *purR* | T686G | Val229Gly |
